# TNFAIP3 Reduction-of-Function Drives Female Infertility and CNS Inflammation

**DOI:** 10.3389/fimmu.2022.811525

**Published:** 2022-04-08

**Authors:** Nathan W. Zammit, Joseph McDowell, Joanna Warren, Walter Muskovic, Joanne Gamble, Yan-Chuan Shi, Dominik Kaczorowski, Chia-Ling Chan, Joseph Powell, Chris Ormandy, David Brown, Samantha R. Oakes, Shane T. Grey

**Affiliations:** ^1^Immunity and Inflammation Theme, Garvan Institute of Medical Research, Sydney, NSW, Australia; ^2^St Vincent’s Clinical School, Faculty of Medicine, University of New South Wales, Sydney, NSW, Australia; ^3^Garvan-Weizmann Centre for Cellular Genomics, Garvan Institute of Medical Research, Sydney, NSW, Australia; ^4^Centre for NSW Health Pathology, Institute of Clinical Pathology And Medical Research, Westmead Hospital, Westmead, NSW, Australia; ^5^Diabetes and Metabolism Division, Garvan Institute of Medical Research, Sydney, NSW, Australia; ^6^Translation Science Pillar, Garvan Institute of Medical Research, Sydney, NSW, Australia

**Keywords:** TNFAIP3, A20, inflammation, reproduction, fertility, neuroinflammation, life-history, evolutionary medicine

## Abstract

Women with autoimmune and inflammatory aetiologies can exhibit reduced fecundity. *TNFAIP3* is a master negative regulator of inflammation, and has been linked to many inflammatory conditions by genome wide associations studies, however its role in fertility remains unknown. Here we show that mice harbouring a mild *Tnfaip3* reduction-of-function coding variant (*Tnfaip3*^I325N^) that reduces the threshold for inflammatory NF-κB activation, exhibit reduced fecundity. Sub-fertility in *Tnfaip3*^I325N^ mice is associated with irregular estrous cycling, low numbers of ovarian secondary follicles, impaired mammary gland development and insulin resistance. These pathological features are associated with infertility in human subjects. Transplantation of *Tnfaip3*^I325N^ ovaries, mammary glands or pancreatic islets into wild-type recipients rescued estrous cycling, mammary branching and hyperinsulinemia respectively, pointing towards a cell-extrinsic hormonal mechanism. Examination of hypothalamic brain sections revealed increased levels of microglial activation with reduced levels of luteinizing hormone. *TNFAIP3* coding variants may offer one contributing mechanism for the cause of sub-fertility observed across otherwise healthy populations as well as for the wide variety of auto-inflammatory conditions to which *TNFAIP3* is associated. Further, *TNFAIP3* represents a molecular mechanism that links heightened immunity with neuronal inflammatory homeostasis. These data also highlight that tuning-up immunity with *TNFAIP3* comes with the potentially evolutionary significant trade-off of reduced fertility.

## Introduction

Autoimmune diseases are more frequent in women than men (1, 2), highlighting the potential impact of autoimmunity on female fecundity. Many autoimmune diseases can directly effect key endocrine and nervous systems (3–5), with thyroid autoimmunity being the most prevalent in women and impacting the hypothalamus. The hypothalamus participates in a hypothalamic–pituitary–gonadal axis essential for the normal menstrual cycle in preparation for fertilisation, egg implantation and embryo development (6). Further to this, women with rheumatoid arthritis (RA), systemic lupus erythematosus (SLE) or multiple sclerosis (MS) can present with a number of fertility indications. These include a reduction in overall fecundity, increased time to pregnancy, as well as reduced birth weight for newborn (7–9).

Reproduction is a highly co-ordinated process that requires tight regulation of pro- and anti- inflammatory processes. Hypothalamic gonadotropin releasing hormone (GnRH) drives the production of luteinising hormone (LH) and follicle stimulating hormone (FSH) from the anterior pituitary which in turn triggers the release of ovarian estrogen and inhibin during the estrous cycle (6). Release of these hormones prepares the ovarian follicle for ovulation and induces decidualisation. This process involves release of pro-inflammatory cytokines and an increase of uterine natural killer cells, which populate the decidua (10). Ovulation itself causes damage to the ovarian surface, with vasculature and tissue remodelling being necessary to transform the follicle into a functional corpus luteum. Progesterone release, promotes uterine decidualisation, a prerequisite for successful embryo implantation (11). A wide variety of cytokines such as IL1, IL6, IL8 and TNF, the synthesis of which are controlled by nuclear factor kappa beta (NF-κB), are crucial mediators of this remodelling process and dysregulation of these inflammatory process can cause failure at any stage of follicle maturation, ovulation, implantation and placentation (11). Thus, the intersection of inflammatory disease with infertility reflects both the interplay between normal immune function and healthy reproductive physiology (3) but potentially a role for common genetic factors.

Human genome wide association scans (GWAS) have linked multiple immune genes with autoimmune disease raising the possibility that inflammatory genetic factors may also influence fertility in disease states. *TNFAIP3* represents a possible inflammatory candidate gene that could influence fertility, as *TNFAIP3* regulates inflammation through control of NF-κB which in turn influences many aspects of fertility, and GWAS data link *TNFAIP3* SNPs with autoimmune diseases with female bias and associated infertility (i.e. with SLE, RA and MS). *TNFAIP3*, encoding A20, is an evolutionarily ancient controller of inflammation (12) – limiting over-zealous activation of innate or adaptive immune cells of the inflammatory response of non-hematopoietic cells through inhibition of NF-κB (12–14). A20 inhibits NF-κB and multiple downstream immune and danger sensing receptors *via* multiple ubiquitin-editing and interacting domains (15–18). A20 expression is regulated both at the level of gene transcription, whereby NF-κB activation induces *TNFAIP3* gene transcription (19, 20) and by post-translational phosphorylation of a key residue Serine-381, which enhances the inhibitory activities of A20 enzymatic sites (21, 22). Thus, *TNFAIP3* is a master negative regulator of NF-κB whose expression is induced during an inflammatory response to restore tissue and immune homeostasis.

The *TNFAIP3* coding SNP F127C decreases *TNFAIP3* anti-NF-κB function (23) and associates with RA, SLE, and MS (23–26). We tested the hypothesis that common *TNFAIP3* genetic variants that subtly reduce the threshold for the proinflammatory NF-κB activation might also influence fertility. For our model of a *TNFAIP3* hypomorphic variant we studied the mouse line carrying the I325N missense variant *(Tnfaip3^I325N^)* that diminishes A20’s anti-NF-κB inhibitory function (13). *Tnfaip3^I325N^* mice subsequently show reduced thresholds of immune cell NF-κB activation, heightened pathogen responses, and evidence of low-grade tissue inflammation that mirrors a subclinical autoimmune phenotype (13, 14). Here we show that carriers of the *Tnfaip3*^I325N^ genotype exhibit impaired fertility, highlighting a molecular mechanism by which common variants in autoimmune risk alleles may link autoimmunity with infertility. These data also highlight that tuning-up immunity with *TNFAIP3* comes with the potentially evolutionary significant trade-off of reduced fertility.

## Results

### Modelling the Impact of Human TNFAIP3 Coding Variants on Fertility With *Tnfaip3*^I325N^ Mice

We have previously reported an A20-hypomorphic mouse line, which carries the I325N missense variant that impairs A20 phosphorylation at ser381 by ~ 50% (13). Reduced A20 phosphorylation subsequently results in a ~40% loss of NF-κB inhibitory function in *Tnfaip3 (Tnfaip3^I325N^)*, resulting in reduced thresholds for both immune and non-immune cell intrinsic NF-κB activation (13, 14). Of interest, mice harbouring the *Tnfaip3^I325N^ allele* exhibit evidence of a subclinical autoimmune phenotype characterised by low-grade pancreas and gut infiltrates that do not progress to overt disease (13). Thus, the I325N *TNFAIP3* variant may be useful to model identified human coding variants for their impact on human reproductive physiology. Human coding variants include F127C, A125V and I207L, that reduce A20’s anti-NF-κB inhibitory activity and associate (GWAS) with human autoimmune and inflammatory disease (13, 23, 26). Furthermore, analysis of human genome data sets (e.g. GnomAD) reveal the presence of a human missense variant at the same I325 position as in the A20-hypomorphic mouse line, highlighting potential for direct relevance of the *Tnfaip3^I325N^* variant for human reproductive physiology.

### *Tnfaip3*^I325N^ Drives Infertility and Impaired Ovarian Function

**Figure 1:**
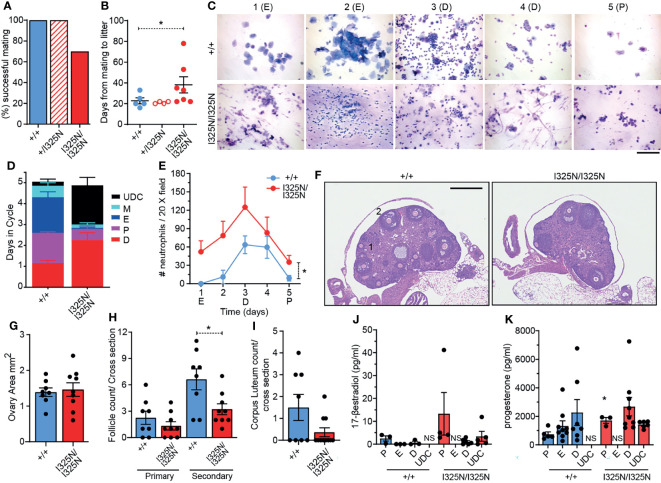
Tnfaip3^I325N^ drives ovarian dysfunction in homozygous mice. Adult (8-12 weeks) female mice of the indicated genotypes were paired with a similar aged wild-type males and **(A)** successful mating and **(B)** time to litter recorded over a 100 day period. (*n* = 5 *Tnfaip3*^+/+^, 4 *Tnfaip3*^+/I325N^ and 10 *Tnfaip3*^I325N/I325N^). **(C)** Diff-Quick stain of vaginal smears over 5 days (D1-D5) from female mice induced to synchronous estrous at 5 weeks of age [Whitten effect (27)]. Data is representative of 1 box of 8 week old mice from 3 independently housed groups (*n* = 5 *Tnfaip3*^+/+^ and 6 *Tnfaip3*^I325N/I325N^). Determined cycle stage for wild-type smears is indicated in parenthesis; E = estrous, D = diesterous, P = proesterous. **(D)** Days spent in each cycle-stage cumulated from all groups in **(C)**. **(E)** The average number of neutrophils identified in a 20X field view of vaginal smears taken in **(C)** Neutrophil count was determined for each slide by numerating and averaging neutrophil number from five random 20X field of views. Counts for 1 full cycle were averaged against counts from 4 additional cycles from the same mouse. **(F)** Representative H&E sections of ovaries from cycling *Tnfaip3* I325N mice of the indicated genotypes. Primary follicles and secondary follicles indicated by 1 and 2 respectively. Scale = 500 µm. **(G)** Ovarian area, **(H)** primary and secondary follicle and **(I)** corpus luteum counts per cross sectional area for *Tnfaip3*^+/+^ and *Tnfaip3*^I325N/I325N^ ovaries. Each point represents an individual ovary. **(J)** 17-β estradiol and **(K)** progesterone ELISA from serum of cycling mice (n ≥ 3). Error bars represent s.e.m and One-Way Anova **(B)**, **(D)** Area under the curve or Student’s T test **(G–K)** used for significance analysis * = P < 0.05. UDC = undefined cycle; NS = no sample.

When paired with wild-type males for 100 days, 30% of *Tnfaip3^I325N/I325N^* females failed to fall pregnant, with those successfully falling pregnant taking on average longer to do so compared to *Tnfaip3^+/+^* and *Tnfaip3^+/I325N^* littermates (i.e. 39 versus 23 days) (**Figures 1A, B**). Despite a longer time to pregnancy, the average number of viable pups per litter was not different between genotypes (e.g. average number of pups per litter = ~4-7 for wild type versus ~4-8 for *Tnfaip3^I325N/I325N^* females). Together these data are indicative of a reduction in fecundity manifested as an increased time to pregnancy which is seen in women with autoimmune disease (7–9). Analysis of cervical swabs of cycling littermates between 5-8 weeks of age showed *Tnfaip3^I325N/I325N^* mice spent many more days in diestrous, or completely failed to enter estrous (**Figures 1C, D** and **Supplementary Table 1**). In addition, swabs of *Tnfaip3^I325N/I325N^* mice contained high numbers of neutrophils at all stages (**Figure 1E**). Because neutrophils are hormonally controlled to mediate the catabolic process of diestrous, these data are indicative of a failure to reach true estrous or exit diestrous and may point towards dysregulated hormonal signalling. In addition, *Tnfaip3*^I325N/I325N^ mice exhibited a significantly reduced number of secondary follicles and a trend towards a decreased number of corpus luteum bodies. Ovarian area and primary follicle numbers were similar between genotype mice (**Figures 1F–I**). As the uterine estrous cycle is controlled by the temporal ovarian release of estrogen and progesterone stimulated by pituitary LH (6, 28), we investigated their serum levels at different stages of the estrous cycle. Subsequently, we found *Tnfaip3*^I325N/I325N^ mice exhibited no difference in serum estradiol levels during bona fide proestrus and diestrus and a significantly increased level of progesterone during proestrus when compared to wild-type littermates. Serum samples taken during undefined cycle (UDC) in I325N homozygotes that typically exhibit features of estrus, proesteros and diestrous (**Figure 1C**) exhibited moderate levels of both hormones. Together these data suggest that ovaries of *Tnfaip3*^I325N/I325N^ mice undergo a period of erroneous cycling characterised by a failure to enter full-esterous associated with dysregulated ovarian sex steroids and corpus luteal remodelling (**Figures 1J, K**). Therefore, these data show that *Tnaip3*^I325N/I325N^ mice exhibit a sub-fertile phenotype. Reduced fecundity compared to their wild-type littermates was characterised by impaired ovarian follicle development, cyclic ovarian function, reduced estrous, and temporal dysregulated release or synthesis of primary sex steroid hormones.

### *Tnfaip3*^I325N^ Mice Exhibit Impaired Mammary Gland Development

**Figure 2:**
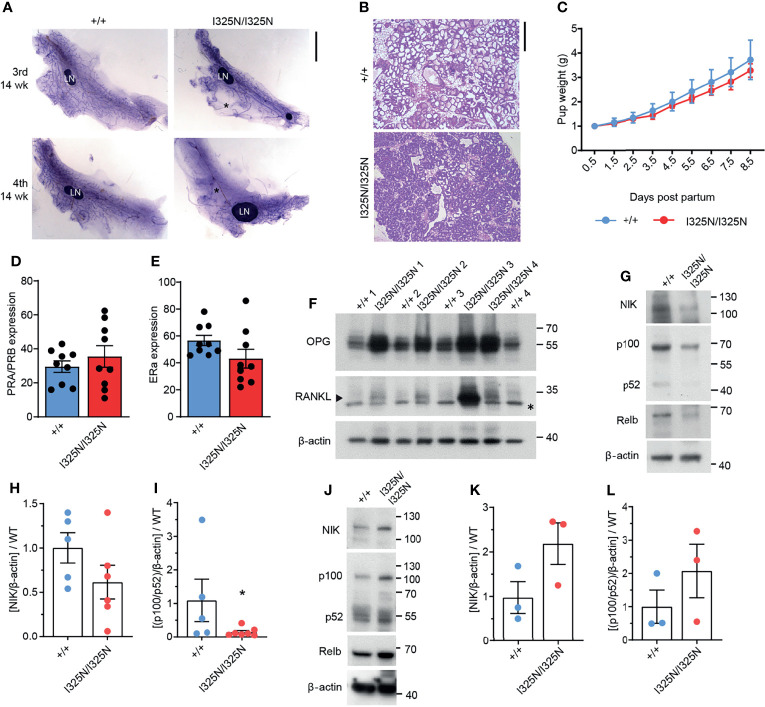
Tnfaip3 I325N mice exhibit impaired mammary development, elevated non-canonical RankL signalling and dampened non-canonical NF-κB signalling. **(A)** Mammary gland whole mounts of the 3^rd^ and 4^th^ mammary gland from 14-week virgin *Tnfaip3*^I325N^ mice of indicated genotype. * denotes empty fat pad space. LN = lymph node. **(B)** Representative H&E of mammary gland from does 8.5 days post-partum, and **(C)** pup weight of resulting pairs, normalised to litter size. **(D, E)** Quantified positive immunohistochemistry staining for **(D)** progesterone receptor (PR) or **(E)** estrogen receptor (ER) (n = 9 *Tnfaip3*^+/+^ and 9 *Tnfaip3*^I325N/I325N^). **(F)** 4^th^ Mammary gland from 10-week-old mice were collected and lysed for immunoblot assessment of paracrine hormones OPG and RANKL with Beta-actin used as the loading control.* denotes non-specific band and arrow head denotes specific band. Molecular weight in Kilodaltons is shown to the right of each blot. **(G–I)** 4^th^ Mammary gland and its lymph node **(J–L)** from 10-week-old mice were collected for immunoblot **(G, J)** and densitometry analysis **(H, I**, **K, L)** for non-canonical NF-κB components NIK, p100/p52 and RelB. Immunoblots are representative of 2 independent experiments with n=6 **(H, I)** or n = 3 **(K, L)** biological replicates for both WT and HOM donors. Densitometry values were normalised to the average WT value of each blot to allow cumulative quantification. Error bars represent s.e.m and Student’s T test used for significance analysis * = P < 0.05.

Ovarian steroid hormones, estrogen and progesterone are crucial for mammary gland development by promoting post-pubertal ductal elongation and side branching, respectively, and for establishment of the lobuloalveoli during pregnancy (29). In mice post-pubertal ductal morphogenesis is complete by 8-14 weeks of age depending on strain. As *Tnfaip3*^I325N/I325N^ mice exhibited dysregulated ovarian cycling, we investigated mammary morphology of adult 14-week old virgin mice. We observed a marked reduction in ductal elongation in the 3^rd^ (axial) and 4^th^ (inguinal) mammary glands of *Tnfaip3*^I325N/I325N^ mice accompanied by grossly enlarged lymph nodes compared to wild-type littermates (**Figure 2A**). Ductal morphogenesis in*Tnfaip3*^I325N/I325N^ was partially rescued by pregnancy where the mammary epithelium completely penetrated the mammary fat pad, but lobuloalveolar development was impaired, characterized by smaller and more compacted lobuloalveoli indicative of attenuated secretory activation or slowed milk secretion into the alveolar luminal space (**Figure 2B**). Despite the impaired lobuloalveolar development, *Tnfaip3*^I325N/I325N^ mice were able to support pup weight gain post-partum (**Figure 2C**). In addition, no significant difference in the proportion of mammary epithelial cells with either estrogen or progesterone receptor expression was detected (**Figures 2D, E** and **Supplementary Figure 1**). Lastly, we next examined for the presence of mammary paracrine hormones RANKL and OPG in 10-week old developing mammary glands. Estrogen stimulates OPG expression (30, 31), and progesterone, RANKL expression, critical for ductal side branching and alveologenesis (32–35). We found *Tnfaip3*^I325N/I325N^ mammary glands to exhibit increased levels of both OPG and RANKL paracrine hormones, compared to *Tnfaip3*^+/+^ littermates (**Figure 2F**), which may fit with the serum presence of both estrogen and progesterone hormones during the prolonged UDC stage of *Tnfaip3*^I325N/I325N^ mice. Together, the above data support a sub-fertility phenotype driven by dysregulated ovarian cycling.

### Decreased Non-Canonical NF-κB Signalling in *Tnfaip3^I325N^* Mammary Glands

The RANKL/RANK/OPG axis directs ductal morphogenesis and pregnancy in response to estrogen and progesterone by fine tuning non-canonical NF-κB mediated transcription of cell cycle mediators such as Cyclin D1 to drive proliferation of the mammary gland (36). RANKL binds RANK receptor (RANK) to activate non-canonical NF-κB, and OPG acts to limit RANKL binding to RANK by binding to RANKL with higher avidity (31, 36). Therefore, we next probed for components of the non-canonical pathway to determine whether observed upregulation of OPG and RANKL in I325N allelic mice resulted in changes in downstream signalling. We observed reduced NIK stabilisation, and decreased levels of P100 to P52 processing (**Figures 2G–I**), indicative of decreased non-canonical NF-κB signalling in *Tnfaip3*^I325N/I325N^ mammary glands. These data conflicts with previous observations in pancreatic islets, where we have reported *Tnfaip*3^I325N/I325N^ islets to exhibit increased non-canonical NF-κB activation (13), which associates with metabolic dysregulation (37). When we examined non-canonical NF-κB signalling in mammary lymph-nodes, we found a trend increase in non-canonical NF-κB activation, highlighting a potential for tissue specific effects (**Figures 2J–L**). Indeed, these data suggest that dysregulated paracrine signalling characterised by high OPG, is the major driver of dampened non-canonical NF-κB activation in the mammary glands of *Tnfaip3*^I325N/I325N^ mice. These data are consistent with previous observations demonstrating that loss of RANKL signalling causes impaired mammary lobuloalveolar development and lactation (38). Together, these data show that the I325N *Tnfaip3* allele drives an impairment in mammary gland development through dysregulated paracrine signalling.

### Cell Extrinsic Cause for Impaired Ovary Function and Mammary Development in *Tnfaip3*^I325N^ Allelic Mice

**Figure 3:**
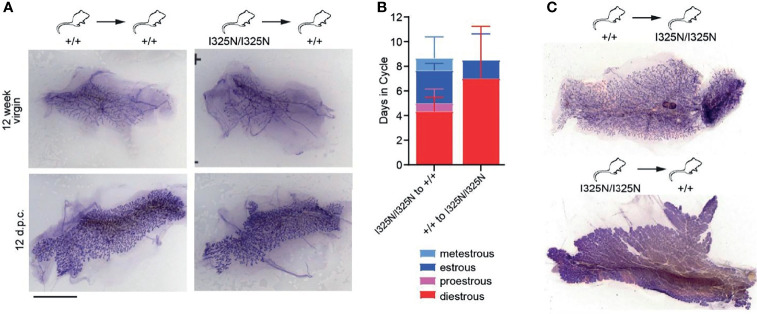
Transplantation of I325N mammary tissue or ovaries into WT recipients rescues mammary development and esterous cycling. **(A)** Representative whole mounts of mammary gland at 8 weeks following mammary epithelial transplants from wild-type (left column) or I325N homozygous (right column) donors into wild-type recipients. Whole mounts were assessed from virgin mice (top row – 12-week virgin) or following pairing with a wild-type male and 12 days post coitum (d.p.c.). Scale bar = 5mm. **(B)** Average days spent in each cycle-stage from wild type mice receiving a *Tnfaip3*^I325N/I325N^ ovary transplant or *Tnfaip3*^I325N/I325N^ mice receiving a wild-type ovary. Diff-Quick stain of cervical smears was to determine cycle-stage. **(C)** Representative whole mount of mammary glands 8 days post-partum of transplanted female7s in **(B)**. Scale bar = 5mm. Error bars represent s.d..

The *Tnfaip3*^I325N/I325N^ allele drives a sub-clinical, low-grade inflammation in the colon, liver and kidneys under SPF conditions, however these mice do not spontaneously develop overt autoimmune or inflammatory disease, (13). As A20 is expressed in ovaries (i.e. https://www.proteinatlas.org/ENSG00000118503-TNFAIP3/tissue) we next assessed the uteri of allelic mice. We observed sub-clinical inflammation evidenced by thickening of the uterine wall and presence of an immune infiltrate along the uterine wall but without overt signs of tissue damage (**Supplementary Figure 2A**). Further to this, we also observed an increased frequency of macrophages (CD45+CD11b+F4/80+ cells), and an increased frequency of ICAM-1+ macrophages compared to allelic null mice (**Supplementary Figures 2B, C**). These data are consistent with a heightened inflammatory state of I325N uteri. As local inflammation represents a barrier to fertility, we next tested whether the effects of *Tnfaip3*^I325N/I325N^ on ovarian function and mammary gland development were cell autonomous. To test this question, we transplanted *Tnfaip3*^I325N/I325N^ mammary epithelial tissue or ovaries into wild-type mice. Transplantation of *Tnfaip3*^I325N/I325N^ mammary epithelial tissue into wild-type hosts rescued post-pubertal mammary ductal elongation and mammary branching morphogenesis during pregnancy (**Figure 3A**; right panel). *Tnfaip3*^I325N/I325N^ mutant mice bearing *Tnfaip3*^+/+^ ovaries showed a similar failed estrous cycle to *Tnfaip3*^I325N/I325N^ mutant mice, but transplantation of *Tnfaip3*^I325N/I325N^ ovaries into ovariectomized wild-type recipients rescued the estrous cycle (**Figure 3B**). Futhermore, wild-type mice bearing *Tnfaip3*^I325N/I325N^ ovaries exhibited fully developed mammary glands 12 days following pregnancy (**Figure 3C**; bottom panel). In contrast, *Tnfaip3*^I325N/I325N^ mice bearing wild-type ovaries exhibited impaired development 12 days following pregnancy (**Figure 3C**; top panel). Together, these data support a cell-extrinsic mechanism for infertility in *Tnfaip3*^I325N/I325N^ mice.

### *Tnfaip3^I325N^* Mice Exhibit Features of Peripheral Insulin Resistance Without Diabetes

Infertility is commonly associated with insulin resistance (39). Following a 16-hour fast *Tnfaip3*^I325N/I325N^ mice exhibited lower blood glucose levels compared to wild-type littermates, but were not hyperinsulinemic (**Supplementary Figures 3A, B**). Similar to previously reported (13), *Tnfaip3*^I325N/I325N^ mice are glucose tolerant following administration of an intraperitoneal or intravenous bolus of glucose (**Supplementary Figures 3C, D**). However, *Tnfaip3*^I325N/I325N^ mice were found to release up to 3 times more insulin in the blood compared to wild-type litter mates in response to the glucose bolus (**Supplementary Figure 3E**), which increased with age (**Supplementary Figures 3F, G**). Hyperinsulinemia is a hallmark of peripheral insulin resistance, typically caused by chronic and systemic inflammation (40–42). Indeed, we previously reported *Tnfaip3*^I325N/I325N^ mice to exhibit marked mononuclear infiltrate within peripheral tissues such as the liver, kidney, colon and pancreas (13), as well as, increased levels of circulating and splenic activated/memory T cells and B cells (13). In addition, macrophages from *Tnfaip3*^I325N/I325N^ mice are more sensitive to activating stimuli (13). Consistent with these data *Tnfaip3*^I325N/I325N^ mice harbour higher frequencies of mononuclear immune cells expressing NF-κB regulated activation markers, including an increased frequency of ICAM1+ splenic macrophages, and an increased frequency of CD44+ T cells in both the spleen and lymph nodes (**Supplementary Figure 4**) (43–45).

To further test the idea of peripheral insulin resistance as the primary mechanism for hyperinsulinemia following glucose challenge in *Tnfaip3*^I325N/I325N^ mice, we isolated and transplanted wild-type islets into *Tnfaip3*^+/+^ or *Tnfaip3*^I325N/I325N^ recipients. Next, we measured blood glucose and insulin output following a glucose bolus in euglycemic mice 14-days post-transplant (**Supplementary Figure 3H**). *Tnfaip3*^I325N/I325N^ recipients harbouring wild-type islets exhibited 3 times higher blood insulin levels compared to wild-type islets transplanted into wild-type recipients (**Supplementary Figure 3J**). These data point to systemic inflammation, extrinsic to the pancreatic beta cell, as the cause for high insulin output following a glucose challenge in *Tnfaip3*^I325N/I325N^ mice. Together, these data illustrate that *Tnfaip3*^I325N^ mice exhibit features for peripheral insulin resistance, however they do not alone explain the observed systemic hormone dysregulation and infertility.

### *Tnfaip3^I325N^* Mice Exhibit Low Levels of Luteinising Hormone Associated With Heightened CNS Inflammation

**Figure 4:**
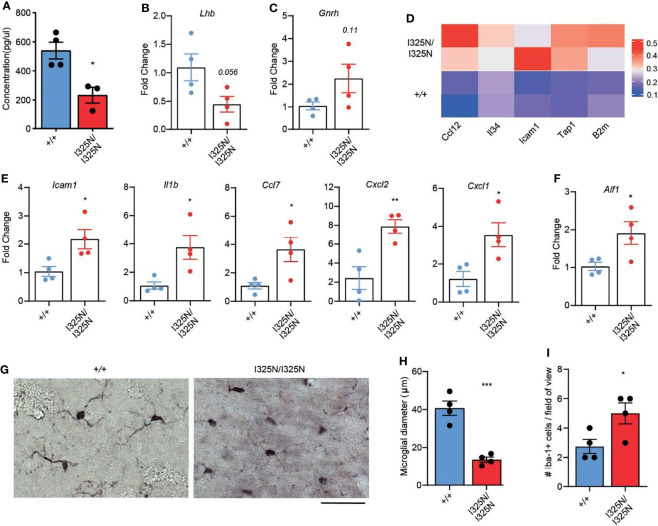
*Tnfaip3^I325N^* mice exhibit reduced serum luteinizing hormone and spontaneous CNS inflammation. **(A)** ELISA for luteinizing hormone from serum plasma from *Tnfap3*^I325N^ littermates of indicated genotypes, and **(B)** RTPCR analysis of brain lysates obtained from dissected pituitary and hypothalamus for luteinizing hormone or **(C)** gonadotropin-releasing hormone mRNA. **(D)** Visium transcriptomic analysis of preoptic area (PO) of *Tnfaip3*^I325N^ littermates of indicated genotypes revealed an upregulation of microglial associated genes at steady state. Each row represents data from an individual mouse. **(E)** RTPCR analysis of brain lysates from dissected pituitary and hypothalamus for microglial-associated inflammatory markers from *Tnfaip3*^I325N^ littermates, and for **(F)** the microglial marker *AIF1* that encodes Iba-1. **(G)** Representative immunohistochemistry images for Iba-1 from *Tnfaip3*^I325N^ littermates of indicated genotypes showing a more ramified phenotype in *Tnfaip3* homozygotes. Scale bar = 20 µm. **(H)** Quantification of microglial diameter and **(I)** number of Iba-1+ cells per field of view from **(F)**. Error bars represent s.e.m and Student’s T test used for significance analysis * = P < 0.05; ** = P < 0.01; *** = P < 0.005.

In addition to ovary-derived sex steroids, cyclic ovarian function is also under the control of CNS hormones, namely luteinizing hormone (LH) emanating from the anterior pituitary (6). We found *Tnfaip3*^I325N/I325N^ mice exhibited a ~ 50% reduction in serum LH compared to *Tnfaip3*^+/+^ littermates (**Figure 4A**). A reduction in serum LH was associated with a decrease in LH-β subunit RNA and a trend towards elevated levels of *Gndh* RNA in hypothalamic-pituitary lysates of *Tnfaip3* allelic mice (**Figures 4B, C**). As LH synthesis and secretion is sensitive to inflammation (46), we assessed the preoptic area that contains neurons responsible for LH release from the pituitary, for the presence of inflammatory factors. We subsequently found only a small subset of genes to be significantly upregulated compared to *Tnfaip3* wild-type littermates (**Figure 4D**). The upregulated genes associated strongly with microglial activation and included antigen presentation genes (Tap1, B2m) (47–49) and activation genes (Ccl12, IL-34 and Icam1) (50–52) that occur in neurodegenerative disease, brain injury and viral infection, and are NF-κB regulated. We next used RTPCR as a more sensitive approach to probe for the presence of other inflammatory mediators within the hypothalamus of *Tnfaip3* littermates, which revealed a significant increase for *Il1β*, *ccl7*, *cxcl2* and *cxcl1* (**Figure 4E**), genes associated with microglial-mediated chronic neurodegeneration (53). Indeed, *Tnfaip3* critically restricts CNS microglia activation and microglia-dependent inflammation (54, 55). RTPCR analysis of *Tnfaip3*^I325N/I325N^ brains revealed an increase in *Aif1* mRNA that encodes the microglial marker Iba-1 (**Figure 4F**). Morphological analysis revealed an increase in activated microglia in the hypothalamus of *Tnfaip3* I325N homozygotes exemplified by increased ramification compared to Iba-1+ cells in the brains of wild-type littermates (**Figures 4G–I**). In contrast, *Tnfaip3*^I325N/I325N^ mice did not exhibit evidence of chronic systemic inflammation as determined by an absence of IL-6 in either the serum or hypothalamus of unstimulated brains (i.e. for *Tnfaip3*^I325N/I325N^ mice versus wild type mice serum levels below detection limit of an IL-6 ELISA; n = ≥ 4 mice per group; data not shown). Together, these data show that the *Tnfaip3* I325N hypomorph causes spontaneous inflammation in the CNS of homozygous mice, associated with a decrease in LH, likely explaining the cell-extrinsic effects (**Figure 3**) on ovarian function, fertility (**Figure 1**) and subsequently mammary gland development (**Figure 2**).

## Discussion

Mammalian reproduction is energetically demanding and to ensure enough energy is available for puberty and the maintenance of reproductive capacity a tight feed-back control network exists, regulated by paracrine, autocrine and neuronal signals (56). The concentration of adipocyte-derived hormone leptin provides information about the amount of available stored energy in the body (57). While the concentration of metabolic hormones insulin and ghrelin, provide ‘real-time’ updates on nutrient availability, absorption and use (56, 58, 59). These signals act at multiple levels, namely the gonads and CNS, were signals are integrated through the complex interactions of the hypothalamic-pituitary-gonadal (HPG) axis (56–59). Together, these systems ensure reproductive success by allowing reproduction to proceed only when conditions are favourable. For example, caloric deficit, stress and insulin resistance can abrogate the HPG axis to promote hypothalamic amenorrhea (60) or polycystic ovary syndrome (PCOS) (61) respectively, and thus down-tune fecundity. In this way finite resources can be temporarily diverted to energetically demanding catabolic processes such as immunity and tissue repair to enhance future reproductive success (62–64). However, how the body genetically tunes these cross-tissue adaptations is still largely unknown.

Here we show that the anti-inflammatory enzyme TNFAIP3 can contribute to the tuning of fecundity. The *Tnfaip3* I325N reduction-of-function allele limits fecundity by driving systemic hormonal dysregulation characterised by reduced serum LH. These changes were associated with increased levels of basal CNS inflammation – as may occur during stress or infection (65, 66). We have previously shown *Tnfaip3* I325N to increase host immunity by lowering NF-κB activating thresholds of T cells, B cells and dendritic cells, achieving superior protective immunity to Coxsackie virus at the expense of microbial tolerance (13). In addition to a loss of microbial tolerance, here we illustrate that fertility is an additional trade-off for increased protective immunity (13, 64).

The *Tnfaip3* I325N allele allows for increased cellular NF-κB activation by impairing A20 function *via* the down-tuning of A20-phosphorylation at ser-381, which enforces A20’s enzymatic activities (21, 22). The severity of the *Tnfaip3* I325N reduction-of-function effect biochemically lies between two additional *Tnfaip3* alleles found in humans that also impair A20 phosphorylation at ser-381 (13). The most severely impaired allele, C243Y, is causative for autosomal Bechets disease (67), while the subtlest, I207L, has been found to beneficially up-tune immunity (13). The I207L allele exhibits a specific population distribution, having been adaptively introgressed into modern peoples of Oceania from ancient Denisovan hominins (13, 68). Other potentially functional *TNFAIP3* coding variants with specific population distributions also exist, namely F127C, A125V and I325V (as identified in GnomAD), raising the potential for subtle genetic tuning of A20 activity to have wide-spread impact on female fertility. In addition, our data would predict that disease associated non-coding variants, as well as more penetrant A20 haploinsufficieny (12, 13, 69) may have significant implications for fertility in inflammatory disease. Thus, by altering immune activation thresholds human *TNFAIP3* genetic variants may contribute to the fertility impairment observed in females with autoimmune disease (7–9). None-the-less, these findings widen the scope of clinical phenotypes impacted by *Tnfaip3* to include fertility.

The transplant studies showing rescue of the I325N ovarian phenotype when placed in wild type hosts (**Figure 3**) support an extrinsic mechanism whereby loss of LH secretion associating with CNS inflammation is sufficient for driving infertility in *Tnfaip3* I325N mice. We also observed a trend to decreased in *Gndh* mRNA in hypothalamic-pituitary lysates of *Tnfaip3* allelic mice, and future studies should investigate this result further by protein analysis. However, TNFAIP3 is expressed by ovaries, suggesting the potential for an ovary intrinsic effect contributing to impaired fertility, indeed intrinsic ovarian inflammation due to the reduced anti-inflammatory activity of the I325N variant may act as an important susceptibility factor following an initial extrinsic trigger (14). Further to this, the transplant data do not rule out an additional contribution by bone marrow derived immune cells that reside within the ovary. Myeloid cells and lymphocytes are activated upon the LH surge to assist proper ovulation. As the *Tnfaip3* I325N variant increases the activation state of host immunity (13) (**Supplementary Figures 2**, **4**) it is probable that hyper-activated and resident ovary immune cells could contribute to improper ovulation and therefore altered ovarian sex-steroid production (70, 71).

Inflammation plays a central role in the metabolic syndrome (40), and neuroinflammation is now considered a central driver for a wide range of neurodegenerative diseases (72, 73) and psychiatric illness’s (74–76). Neuroinflammation may also explain clinical links between autoimmune disorders and neuropsychiatric disease (77). Indeed, *Tnfaip3* a central immune regulator, is linked to increased susceptibility to many inflammatory and autoimmune diseases (12, 69), and has recently been found to play a central protective role for the maintenance of CNS homeostasis. *Tnfaip3* deficiency causes spontaneous CNS inflammation characterised by microglial activation and induction of inflammatory mRNAs in the brain (54). A20 is highly expressed in microglia (78), the resident immune cell of the brain (79), and its deficiency within microglia drives a potent inflammatory response, triggering the infiltration of CD8 T-cells and neurodegenerative disease (80, 81). Here we observe increased *Aif1* mRNA and Iba-1 positive microglia with an ‘activated’ morphometry in the brains of *Tnfaip3* I325N mice. These data are consistent with the hyper-inflammatory state of peritoneal macrophages harbouring the same *Tnfaip3* allele (13) or with A20 deficiency (24). Given microglia’s important role in synapse formation, synapse pruning as well as neural myelination (82–84) all important for CNS signal transmission and function, loss of A20 function may impact or predispose to a wide range of neurodegenerative disease including neuropsychiatric conditions (85), as well as fertility as described here.

Neuroinflammation has been suggested to drive infertility by disrupting the hypothalamic-pituitary-gonadal axis, through yet completely understood mechanisms. One potential mechanism is through driving insensitivity of CNS insulin receptors to insulin, termed insulin resistance. Similar to our study, female mice with CNS-specific KO of insulin receptor exhibited ovarian dysfunction and reduced serum and RNA levels of LH (58). Indeed, mice with the *Tnfaip3* I355N allele exhibit peripheral insulin resistance and CNS inflammation, thus identifying a shared mechanism of infertility. A role for inflammatory driven insulin resistance that is independent to obesity induced hormonal dysregulation, such as of leptin, was demonstrated in a transgenic line of lean normoglycemic mice with insulin resistance that exhibited altered duration of estrous cycle and dysfunctional ovarian follicles (86). Lastly, conditions such as Type A syndrome and lipoatrophic diabetes that are characterised by severe insulin resistance, exhibit perturbed hypothalamic-pituitary-gonadal function and ovarian dysfunction (87, 88).

These data underline a novel role for *Tnfaip3* in the maintenance of fertility. Future studies would focus on determining whether neuronal inflammation is causal to the sub-fertility impact of the *Tnfaip3* I355N allele, and the full nature of the fecundity defect including whether it involves a delay in puberty. As NF-κB controls male mouse sperm counts (89) these data also highlight a mechanism for *TNFAIP3* to regulate male fertility. Finally, *TNFAIP3* coding variants have been identified with population distinct distributions. It has been hypothesised that common genetic variants with medium to low penetrance may play an important role in influencing infertility, which is globally wide-spread and common (~5-25% of the female population), yet not well explained (90–92). *TNFAIP3* coding variants may offer one mechanism for the cause of sub-fertility observed across otherwise healthy populations as well as for the wide variety of auto-inflammatory conditions to which *TNFAIP3* is associated. Therapies targeting pathways and inflammatory products normally controlled by *TNFAIP3* may serve as promising treatments to restore the functioning of hypothalamic-pituitary axis and fertility.

## Methods

### Animal Models

The *Tnfaip3 lasvegas* strain (*Tnfaip3*^I325N^) was generated by *N*-ethyl-*N*-nitrosourea (ENU) mutagenesis in C57BL/6 mice, and propagated by backcrossing to C57BL/6 (13, 14). The strain was maintained as heterozygote breeding pairs so that wild type littermates could be used for controls. C57BL/6 and NOD.Cg-Prkdc^scid^ Il2rg^tm1Wjl^/SzJ mice were purchased from Australian Bioresources. Animal studies were conducted in compliance with guidelines of the Garvan Institute Animal Ethics Committee.

### Mating Studies

Adult female wild type and *Tnfaip3*^I325N^ variant mice (8-12 weeks of age) were paired with wild type male mice, and pregnancy was confirmed by the observation of a cervical plug. For fecundity studies n = 5 wild type, n = 4 *Tnfaip3*^I325N/+^ and n = 6 *Tnfaip3*^I325N/I325N^ mice were examined. The onset to pregnancy for *Tnfaip3*^I325N^ variant mice was significantly delayed as shown in Results, but the average number of viable pups per litter was not significantly different between genotypes (average 4-5 pups per litter).

### Monitoring Estrous Cycling

5-week-old *Tnfaip3* mice were monitored for the stage of the estrous cycle by gentle daily vaginal smearing and staining with Diff-Quik (ProSciTech) Romanowsky stain as per the manufacturers instructions.

### Mammary Whole Mount

Mammary whole-mounts were performed using the Carmine alum technique as described before (93). Briefly, at experimental time points, mice were euthanised with CO2 asphyxiation and cervical dislocation and mammary glands harvested and spread onto Superfrost plus glass slides and placed into 10% neutral buffered formalin overnight at room temperature. Mammary wholemounts were defatted with at least 4 changes of acetone and then rehydrated before staining in a 0.2 w/v solution of Carmine alum containing potassium aluminium sulphate and thymol. Mammary wholemounts were then dehydrated with graded alcohols before clearing in Slightbright for 1hr at RT and storage in methylsalycilate. Wholemounts were photographed on a Leica DMRB light microscope and imaged using a Leica DC200 camera.

### Tissue Transplants

Mammary epithelial transplants were performed exactly as previously described (94). Note that to determine whether the mammary phenotype was cell autonomous, either *Tnfaip3*^I325N^ tissue or wild type tissue was transplanted into NOD.Cg-Prkdc^scid^ Il2rg^tm1Wjl^/SzJ recipients. Ovarian transplants were performed on 3-4-week-old donor and recipients. Mice were paired and anaesthetized simultaneously with 75mg/kg ketamine and 0.75mg/kg medetomidine IP. A small incision was made in the left and right dorsal flanks of each mouse perpendicular to the spine and across the abdomen just caudal to the last rib and the abdomen was opened. The ovarian fat pad was grasped and externalised onto a sterile gauze so that a small incision could be made in the ovarian bursa and the ovary dissected free. Pressure was placed on the ovarian stalk for a 20-30 secs using forceps to stop any bleeding. Each donor ovary was then placed in in the left and right empty ovarian bursas of recipient mice and the ovary and uterus re-internalised into the abdomen. The abdominal wall and skin closed with silk sutures and recipient mice monitored for estrous cycling from 5 weeks of age.

Islets were isolated as previously described (95), and counted for islet transplantation using a Leica MZ9.5 stereomicroscope. Islets were transplanted under the kidney capsule of diabetic C57BL/6 littermates as described (96, 97). Diabetes was induced by intraperitoneal injection of 180 mg/kg streptozotocin (Sigma-Aldrich) dissolved in 0.1 M citrate buffer (pH 4.2) at a concentration of 20 mg/ml. Diabetes was determined as [blood glucose] ≥16 mM on two consecutive days measured by FreeStyle Lite^®^ glucometer and Abbott Diabetes Care test strips following tail tipping.

### Flow Cytometry

Flow cytometric staining was performed as described (13). For mouse lymphocytes, fluorochrome-conjugated antibody clones against the following surface antigens were: CD4 (RM4-5), CD44 (IM7), CD62L (MEL-14), CD45.2 (104), CD11b (M1/70), F4/80 (RUO), ICAM1 (HCD54). Antibodies were purchased from BD, eBioscience or BioLegend. Data were acquired with LSR II and FORTESSA flow cytometers (BD) and analysed using FlowJo software (Tree Star).

### Metabolic and Hormonal Analysis

Blood glucose levels were determined using a FreeStyle Lite^®^ glucometer and blood glucose test strips (Abbott Diabetes Care) *via* tail tipping. Intraperitoneal glucose tolerance tests (IP-GTT) were conducted following an overnight fast (16 h) with access to water. The following day mice were weighed and fasting blood glucose measurements taken. Subsequently, mice were injected intraperitoneally with 2 g/kg 20% dextrose (Sigma Aldrich). Blood glucose levels were measured from the tail vein at 15, 30, 60 and 120 min post-dextrose administration. Intravenous (IV) GTT was conducted in a similar manner; however, glucose (1 g/kg) was administered intravenously into the tail vein and blood glucose measurements taken at 0, 5, 10, 15, 20, 30, and 60 min post-injection. During the IV-GTT blood samples were also taken for the determination of insulin content *via* ELISA, conducted as per the manufacture’s instructions (Cayman Chemical). Serum ELISA for 17β-estradiol (Cayman Chemical; 501890), progesterone (Cayman Chemical; 582601) and Luteinizing hormone (Elabscience; E-EL-M3053) were performed as per manufacturers’ instructions

### Immunohistochemistry

Ovarian tissues were fixed in 10% neutral buffered formalin (Sigma-Aldrich), paraffin embedded and parallel sections (5-10 µm) prepared. Sections were stained with hematoxylin and eosin (H&E Sigma-Aldrich). Images were captured using a Leica DM 4000 or Leica DM 6000 Power Mosaic microscope (Leica Microsystems). Quantification of the number of follicles per cross sectional area was conducted by taking the average from 5 serial sections separated by 500 µm each. Progesterone and estrogen receptor staining was performed as previously described (98). Briefly, formalin fixed paraffin embedded sections were dewaxed, dehydrated and antigen retrieval performed with pH6 retrieval solution (Dako S1699) in a pressure cooker at 125°C for 30 sec. Endogenous peroxidase was blocked by 3% H2O2 and sections were stained with an antibody to ERα (Santa Cruz MC20 SC-542) or PRA and PRB (Dako A0098) for 30 mins at RT. The sections were then incubated with Envision rabbit HRP secondary (Dako K4002) for 30 min at RT prior to application of 3,3’Diaminobenzidine plus tertiary substrate (Dako K3468) for 10 mins at RT. Image quantification of the percentage positive epithelial nuclei was performed using macros designed for the FIJI image analysis software (http://fiji.sc/Fiji). The percentage positive of 3 representative images from each animal (5 per group) is shown in **Figures 2D, E**.

CNS tissue was examined by IHC. Briefly, mice were deeply anesthetized (350mg/kg pentobarbitone sodium) and perfused through the left ventricle with 100 ml 0.9% saline, followed by 150 ml ice-cold 4% paraformaldehyde in sodium borate buffer at pH 9.5 using a peristaltic pump (Gilson). Brains were harvested, postfixed in 4% paraformaldehyde for 5 hours, and cryoprotected for 16 hours in 20% sucrose/50 mM potassium PBS (KPBS) at 4°C. Next, 30-μm-thick frozen brain sections were obtained with a sliding microtome (SM2010R; Leica Biosystem). Sections were stored in cryoprotectant solution (30% ethylene glycol, 20% glycol in 50 mM sodium phosphate buffer, pH 7.4) at –20°C. Next, 30-μm-thick floating tissue sections were treated with 0.3% hydrogen peroxide for 10 minutes to inhibit endogenous peroxidase, followed by 7 minutes of 1% sodium borohydride.

Tissue sections were then immunostained with monoclonal antibody against Iba-1 (rabbit anti-mouse Iba-1:1,000; abcam AB108539) in 2% donkey serum and 0.3% Triton X-100. Tissue sections were incubated in primary antibody for 48 hours at 4°C on an orbital rocker (50 rpm; Ratek Instrument), followed by biotinylated secondary staining (donkey anti-rat 1:200; Jackson ImmunoResearch) in the same buffer as above for 1 hour at room temperature. After secondary immunostaining, sections were labelled with avidin using the Vecta kit (Vector Laboratories) per the manufacturer’s protocol. Sections were then washed twice (10 minutes per wash) with KPBS, and staining was developed in DAB without nickel enhancement (0.5 mg/ml DAB, 2 mg/ml D β-glucose, 0.4 mg/ml ammonium chloride, 1 U/ml glucose oxidase; Sigma-Aldrich) in KPBS to give brown labelling. Sections were mounted onto slides in an aqueous mounting buffer and coverslipped. Iba-1 positive microglia where quantified using random fields of view from brain sections across four levels. Microglial diameter was assessed by measuring the diameter of the boundary circle using Image J.

### Immunoblot Analysis

Mammary and lymphoid tissue were collected in Cell Lysis Buffer (Cell Signalling Technology), supplemented with EDTA-free protease inhibitor 1045 [Roche]). Protein concentration was measured using the Bradford assay (Bio-Rad) and total protein (20-25 µg) resolved on a 7 – 10% SDS PAGE gel and then transferred to a nitrocellulose membrane, Immobilon-P^®^ (Merck Millipore). Non-cannonical NFκB pathway activation was assessed as described (37). Briefly, membranes were incubated anti-OPG (SANTA CRUZ E-10; Sc-390518), anti RANKL (R&D AF462), anti NIK (4994), anti-NF-kB2 P100/P52 (4882), anti-Relb (C1E4, 4922) (Cell signalling Technology), and anti-beta-actin (AC15) (Sigma-Aldrich), followed by horseradish peroxidase (HRP)-labelled secondary antibody goat-anti mouse IgG Fc (Pierce Antibodies) or donkey-anti-rabbit IgG (GE Life Sciences). HRP conjugates bound to antigen were detected and visualized by using an ECL detection kit (GE Life Sciences). Densitometry analysis was conducted using ImageJ software (https://imagej.nih.gov/ij/).

### Visuim Transcriptomics

Brains from euthanised mice were set in OCT blocks and snap frozen in liquid nitrogen-chilled isopentane. Individual brains were then and stored at minus 80 until use. For cryosectioning, both the tissue block and the Visium slide were equilibrated inside the cryostat for 15-30 min. Next using an Allen Brain atlas 10 um coronal sections made to capture the medial preoptic area (MPO; Figure 29). Array slides containing sections were stored at minus 80 for a maximum of 1 week before use. Samples were processed according to the Visium Spatial Gene Expression User Guide (10× Genomics) and all reagents were from the Visium Spatial Gene Expression Kit (10× Genomics). Sections were first methanol fixed, stained with H&E and imaged at 10X using a Leica DM6000 microscope. Slides were then processed using an optimised permeabilization time of 9 min (determined using the Visium Spatial Tissue Optimization Kit). The resulting cDNA library was checked for both quality and quantity, and sequenced using an Illumina NovaSeq6000 system using the recommended parameters at the recommended depth, based on slide area occupancy.

### Visium Spatial Transcriptomics Data Processing

Reads were demultiplexed and mapped to the mouse reference genome GRCm38 using the Space Ranger software v.1.1.0 (10x Genomics). Count matrices were analysed with Seurat v.4.0.0 for all subsequent data filtering, normalization, dimensional reduction and visualization. Data normalization was performed on independent tissue sections using the variance-stabilizing transformation method implemented in the Seurat function SCTransform. Voxels corresponding to the medial preoptic area (MPO) were manually defined following expert annotation. Mean normalised expression values of microglial associated genes were quantified across the MPO of each section and visualised as a heatmap.

### Real Time Quantitative PCR

Total RNA was extracted from brain tissue using the RNeasy Plus Mini Kit (Qiagen) and reverse transcribed using Quantitect Reverse Transcription Kit (Qiagen). PCR reactions were performed on the LightCycler^®^ 480 Real Time PCR System (Roche) using the TaqMan system (Thermofisher; **Supplementary Table 2**). Cyclophilin (CPH2) and ACTB were used as housekeeping genes and data analysed using the 2ΔΔCT method. Data were normalized to average WT value. Initial denaturation was = performed at 95°C for 10 sec, followed by a three-step cycle consisting of 95°C for 15 sec = (4.8°C/s, denaturation), 63°C for 30 sec (2.5°C/sec, annealing), and 72°C for 30 sec (4.8°C/s, elongation). A melt-curve was performed after finalization of 45 cycles at 95°C for 2 min, 40°C for 3 min and gradual increase to 95°C with 25 acquisitions/°C.

### Statistics

All data are presented as mean ± s.e.m. Student’s *t*-test or 1- or 2- way ANOVA analysis were performed, depending on experimental design, to determine statistical difference between groups. A P value less than 0.05 was considered significant. Tests were conducted on Prism (v8) software (GraphPad Software).

### Study Approval

All procedures involving animals were carried out according to the guidelines established by the Australian Institutional Animal Ethics Committee guidelines. Animal studies were approved by the Garvan/St Vincent’s Animal Ethics Committee. All procedures performed complied with the Australian Code of Practice for Care and Use of Animals for Scientific Purposes.

## Data Availability Statement

The original contributions presented in the study are included in the article/**Supplementary Material**. Further inquiries can be directed to the corresponding authors.

## Ethics Statement

The animal study was reviewed and approved by Garvan Institute Animal Ethics Committee.

## Author Contributions

Experimental analysis of ovarian dysfunction in *Tnfaip*^3I355N^ mice conducted by NZ, SO, CO, and JM. Mammary gland development studies conducted by NZ, CO, and SO. Ovary transplantation studies performed by CO and SO. Islet transplant and metabolic studies performed by NZ. ELISA studies conducted by JW. Brain gene expression analysis by NZ and JW. Brain pathology analysis JG and DB. Visium sample preparation conducted by JW, YS, and NZ. Visium analysis conducted by WM, DK, CC, JP and NZ. Experimental design and analysis performed by NZ, SO, and SG. Manuscript preparation by NZ, SO, and SG. SG led the study and is the study guarantor. All authors contributed to the article and approved the submitted version.

## Funding

The research was supported by grants to SG from the NIH (DK076169) and NHMRC (GNT1130222; GNT1189235), a NHMRC Fellowship to CJO (481310), a National Breast Cancer Foundation Fellowship to SRO (ECF-13-08; ECF-16-022), and an Australian Postgraduate Award and International Pancreas and Islet Transplant Association (IPITA) Derek Gray Scholarship to NZ. SG is supported by an NHMRC Senior Research Fellowship Level B (GNT1140691).

## Conflict of Interest

The authors declare that the research was conducted in the absence of any commercial or financial relationships that could be construed as a potential conflict of interest.

## Publisher’s Note

All claims expressed in this article are solely those of the authors and do not necessarily represent those of their affiliated organizations, or those of the publisher, the editors and the reviewers. Any product that may be evaluated in this article, or claim that may be made by its manufacturer, is not guaranteed or endorsed by the publisher.

## Supplementary Material

Supplementary Figure 1 Representative photomicrograph of estrogen receptor (ERa) or progesterone receptor (PRA/PRB) stained sections of mammary glands from mice of the indicated genotypes (n = 5 *Tnfaip3*^+/+^ and 5 *Tnfaip3*^I325N/I325N^).

Supplementary Figure 2 **(A)** Representative H&E of uterine walls of mice with or without the I325N allele. Scale bar for paired low (left column) and high power (right column) images = 500 and 200 µm respectively. **(B, C)** Flow cytometric analysis of digested uteri showing the frequency of infiltrating cells that are **(B)**, CD45+ CD11b+ F4/80+ cells, and which are defined as macrophages and **(C)**, ICAM1+ macrophages (CD45+ CD11b+ F4/80+ cells) **(B)**. * = area of high immune infiltrate.

Supplementary Figure 3 A20 I325N mice produce high levels of insulin following a glucose challenge and exhibit peripheral insulin resistance. **(A)** Fasting glucose and **(B)** insulin levels of chow fed 16-week-old female mice with the indicated genotypes. **(C, D)** Blood glucose levels (BGL) were monitored following an **(C)** intraperitoneal (IP; 2 g/kg) (+/+ n = 10; I325N/+ n = 14; I325N/I325N n = 8) or **(D)** intravenous injection (IV; 1 g/kg) of glucose (+/+ *n* = 6; I325N/+ *n* = 3; I325N/I325N *n* = 5). **(E)** Blood insulin levels (ng/ml) were measured following intravenous injection in **(D)**. Blood insulin levels following IVGTT was also measured in 32 **(F)** and 52 **(G)** week old female mice. **(H–J)** To test if A20 I325N mice exhibit peripheral insulin resistance, wild-type (+/+) islets were isolated and transplanted into age and weight-matched streptozotocin-induced diabetic syngeneic recipients that were homozygous, or null for the I325N variant (i.e. WT -> WT *n*=4; WT -> HOM *n* = 4 [donor -> recipient]). **(I)** Following transplantation blood glucose levels (BGLs) were monitored for 13 days. **(I)** At post-operative day (POD) 14 glucose tolerance was tested *via* IV injection of a glucose bolus (1g/kg). **(J)** Blood insulin levels (ng/ml) were measured *via* ELISA from blood samples taken. Statistical significance calculated by area under the curve analysis followed by student’s *t* test; * = P < 0.05; error bars ± SEM.

Supplementary Figure 4 The frequency of CD4+ effector memory cells in the **(A)**, Spleen; **(B)**, LN; and **(C)**, peripheral blood. Frequency of macrophages (CD45+ CD11b+ F4/80+ cells) in the **(D)** spleen; and, frequency of ICAM1+ macrophages in the spleen **(E)**. Error bars represent S.E.M and Student’s T test used for significance analysis * = P < 0.05; ** = P < 0.01.

Supplementary Table 1 Ovarian cycling was assessed from 5-8 weeks of age and monitored weekly. Note the homozygous I325N mice did not cycle in early stages, then ultimately became pregnant but also with a delay i.e. time to pregnancy for I325N mice = 38.7 days versus 22.8 days for wild type mice.
