# Supplementary material for: TNFAIP3 Reduction-of-Function Drives Female Infertility and CNS Inflammation
Source: Front Immunol. 2022 Apr 8;13:811525. doi: 10.3389/fimmu.2022.811525 (PMC9027572; doi:10.3389/fimmu.2022.811525)
Supplement: Supplementary Figure 1 — Representative photomicrograph of estrogen receptor (ERa) or progesterone receptor (PRA/PRB) stained sections of mammary glands from mice of the indicated genotypes (n = 5 Tnfaip3+/+ and 5 Tnfaip3I325N/I325N). [file DataSheet_1.docx]

**Supplementary Files**

**TNFAIP3 reduction-of-function drives female infertility and CNS inflammation**

Nathan W. Zammit^1,2¥^, Joseph McDowell^1,2^, Joanna Warren^1,2^, Walter Muskovic^2,3^, Joanne Gamble^4^, Yanchuan Shi^5^, Dominik Kaczorowski^2,3^, Chia-Ling Chan^2,3^, Joseph Powell^2,3^, Chris Ormandy^2,6^, David Brown^4^, Samantha R. Oakes^2,6^, Shane T. Grey^1,2,6¥^

**Affiliations:**

^1^Immunity & Inflammation Theme, Garvan Institute of Medical Research, Darlinghurst, NSW, 2010 Australia

^2^St Vincent’s Clinical School, Faculty of Medicine, University of New South Wales Sydney, NSW 2010, Australia

^3^Garvan-Weizmann Centre for Cellular Genomics, Garvan Institute of Medical Research, Darlinghurst, NSW, Australia

^4^Centre for NSW Health Pathology, Institute of Clinical Pathology And Medical Research, Westmead Hospital, Westmead 2145, NSW

^5^Diabetes and Metabolism Division, Garvan Institute of Medical Research, Darlinghurst, NSW, Australia,

^6^Translation Science Pillar, Garvan Institute of Medical Research,

Darlinghurst, NSW, Australia,

Correspondence to: N.W.Z [n.zammit@garvan.org.au](mailto:n.zammit@garvan.org.au) or S.T.G [s.grey@garvan.org.au](mailto:s.grey@garvan.org.au)

**Running Title:**

TNFAIP3 trades-off inflammation for fertility

**Key Words:**

TNFAIP3, A20, Inflammation, Reproduction, Fertility, Autoimmunity, Neuroinflammation, Trade-off, Evolution, Immunity


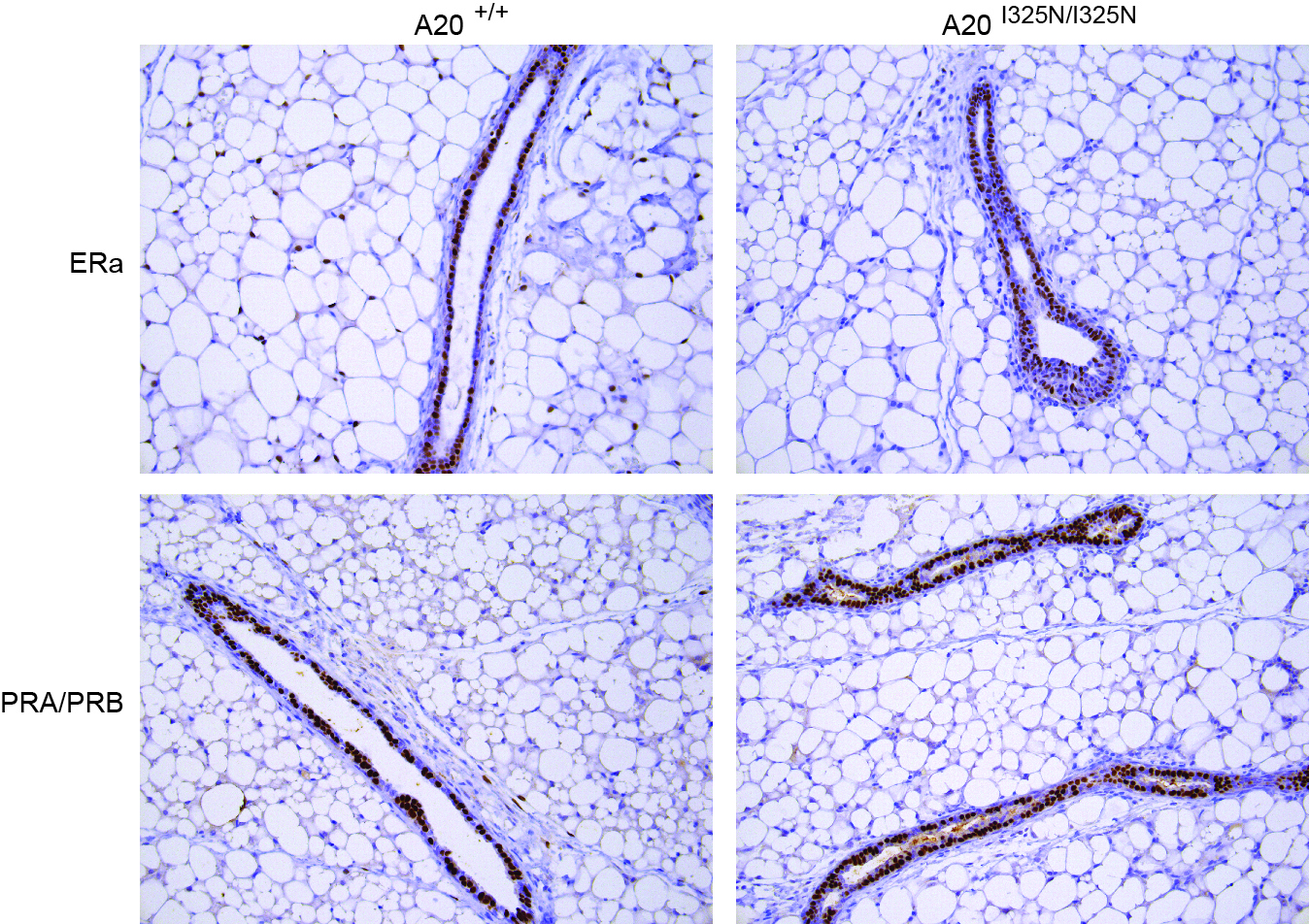


**Supplementary Figure 1** Representative photomicrograph of estrogen receptor (ERa) or progesterone receptor (PRA/PRB) stained sections of mammary glands from mice of the indicated genotypes (n=5 *Tnfaip3*^+/+^ and 5 *Tnfaip3*^I325N/I325N^).


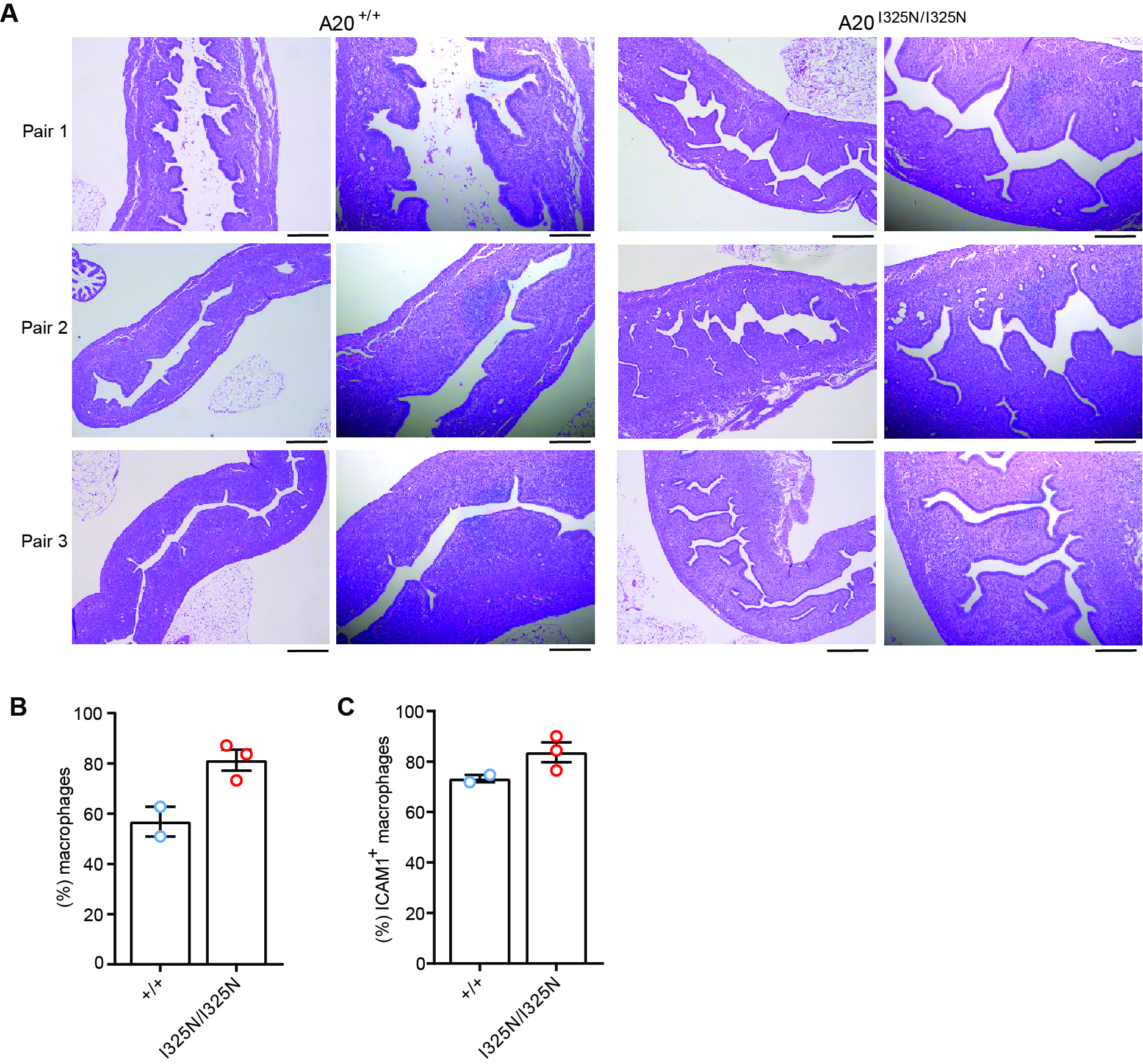


**Supplementary Figure 2**

**A)** Representative H&E of uterine walls of mice with or without the I325N allele. Scale bar for paired low (left column) and high power (right column) images = 500 and 200 µm respectively.
**B-C)** Flow cytometric analysis of digested uteri showing the frequency of infiltrating cells that are (B), CD45+ CD11b+ F4/80+ cells, and which are defined as macrophages and (C), ICAM1+ macrophages (CD45+ CD11b+ F4/80+ cells) (B). * = area of high immune infiltrate.


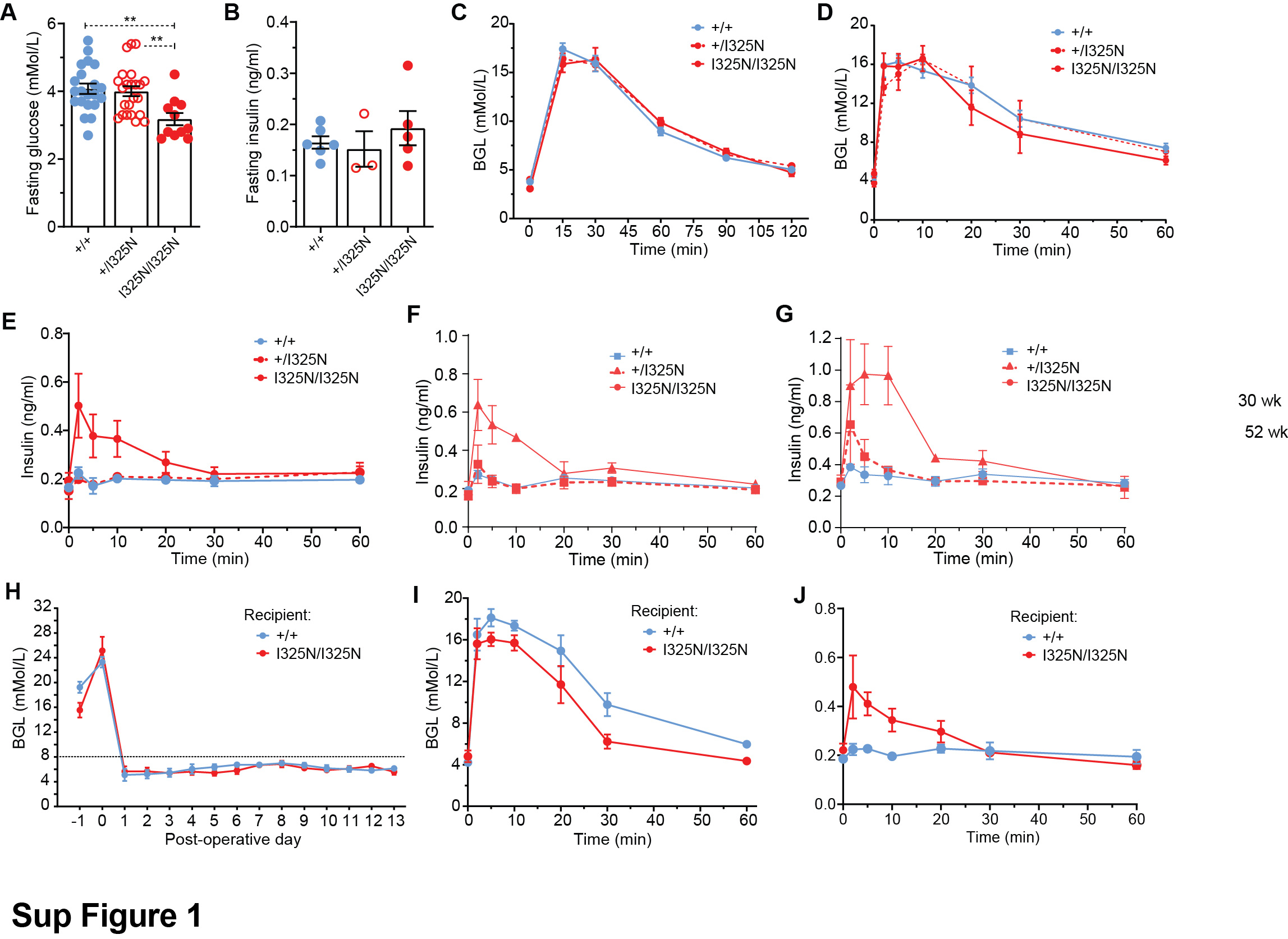


**Supplementary Figure 3**

A20 I325N mice produce high levels of insulin following a glucose challenge and exhibit

peripheral insulin resistance. **(A)** Fasting glucose and **(B)** insulin levels of chow fed 16

week-old female mice with the indicated genotypes. **(C-D)** Blood glucose levels (BGL)

were monitored following an (C) intraperitoneal (IP; 2 g/kg) (+/+ n=10; I325N/+ n=14;

I325N/I325N n=8) or (D) intravenous injection (IV; 1 g/kg) of glucose (+/+ *n*=6; I325N/+

*n*= 3; I325N/I325N *n*=5). **(E)** Blood insulin levels (ng/ml) were measured following

intravenous injection in (D). Blood insulin levels following IVGTT was also measured in

32 **(F)** and 52 **(G)** week old female mice.

**(H-J)** To test if A20 I325N mice exhibit peripheral insulin resistance, wild-type (+/+)

islets were isolated and transplanted into age and weight-matched streptozotocin

induced diabetic syngeneic recipients that were homozygous, or null for the I325N

variant (i.e. WT -> WT *n*=4; WT -> HOM *n*=4 [donor -> recipient]). **(I)** Following

transplantation blood glucose levels (BGLs) were monitored for 13 days. **(I)** At post

operative day (POD) 14 glucose tolerance was tested via IV injection of a glucose bolus

(1g/kg). **(J)** Blood insulin levels (ng/ml) were measured via ELISA from blood samples

taken. Statistical significance calculated by area under the curve analysis followed by

student’s *t* test; * = P<0.05; error bars ± SEM.

**
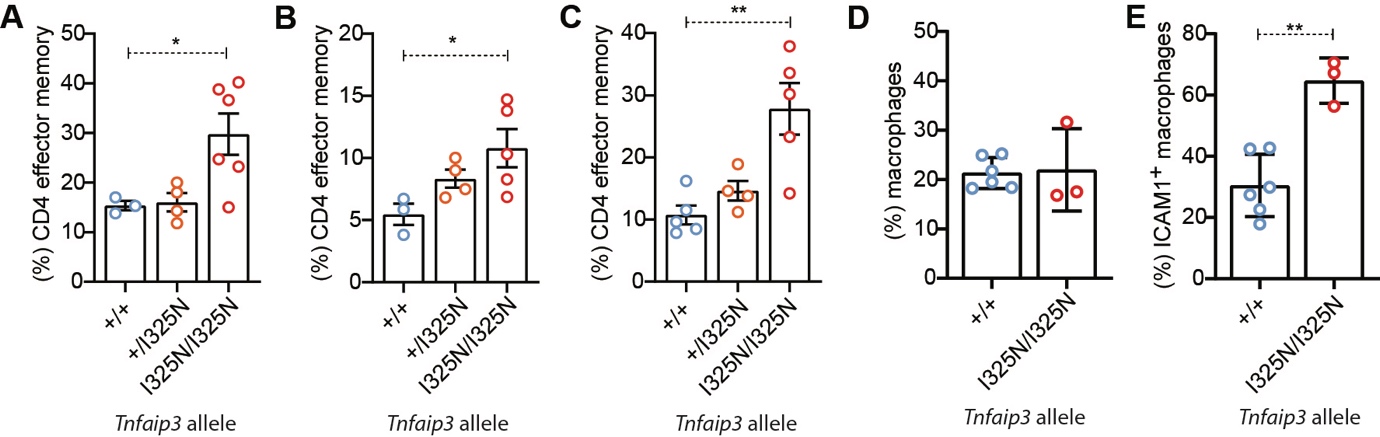
**

**Supplementary figure 4**

The frequency of CD4+ effector memory cells in the (A), Spleen; (B), LN; and (C), peripheral blood. Frequency of macrophages (CD45+ CD11b+ F4/80+ cells) in the (D) spleen; and, frequency of ICAM1+ macrophages in the spleen (E). Error bars represent S.E.M and Student’s T test used for significance analysis * = P<0.05; ** = P<0.01.

**Supplementary table 1**Ovarian cycling was assessed from 5-8 weeks of age and monitored weekly. Note the homozygous I325N mice did not cycle in early stages, then ultimately became pregnant but also with a delay i.e. time to pregnancy for I325N mice = 38.7 days versus 22.8 days for wild type mice.

|  |  | Week | | | |
| --- | --- | --- | --- | --- | --- |
| Number of mice in cycle | Cycling | 5 | 6 | 7 | 8 |
| 5 | WT | 3 | 4 | 5 | 5 |
| 2 | HET | 1 | 2 | 2 | 2 |
| 6 | HOM | 0 | 0 | 0 | 1 |
|  |  |  |  |  |  |
| Percent cycling |  |  |  |  |  |
| 5 | WT | 60.00% | 80.00% | 100.00% | 100.00% |
| 2 | HET | 20.00% | 40.00% | 40.00% | 40.00% |
| 6 | HOM | 0.00% | 0.00% | 0.00% | 20.00% |

**Supplementary table 2**

| Description | Catalogue Number | Assay ID |
| --- | --- | --- |
| mouse Gnrh1 | 4331182 | Mm01315604_m1 |
| mouse Lh | 4331182 | Mm00656868_g1 |
| mouse Cxcl10 | 4331182 | Mm00445235_m1 |
| mouse TNF | 4331182 | Mm00443258_m1 |
| mouse IL1B | 4331182 | Mm00434228_m1 |
| mouse ICAM1 | 4331182 | Mm00516023_m1 |
| mouse CCL2 | 4331182 | Mm00441242_m1 |
| mouse ionized calcium-binding adapter molecule 1 (Iba1) | 4331182 | Mm00479862_g1 |
| mouse tnfaip3 | 4331182 | Mm00437121_m1 |
